# Supplementary material for: High-speed tunable microwave-rate soliton microcomb
Source: Nat Commun. 2023 Jun 12;14:3467. doi: 10.1038/s41467-023-39229-3 (PMC10260980; doi:10.1038/s41467-023-39229-3)
Supplement: Supplementary file 1 — Supplementary Information [file 41467_2023_39229_MOESM1_ESM.pdf]

"High-speed tunable microwave-rate soliton microcomb" Supplementary Information

Yang He,[1,*] Raymond Lopez-Rios,[2,*] Usman A. Javid,[2] Jingwei Ling,[1] Mingxiao Li,[1] Shixin Xue,[1] Kerry Vahala,[3] and Qiang Lin[1,2,†]

[1]*Department of Electrical and Computer Engineering, University of Rochester, Rochester, NY 14627*
[2]*Institute of Optics, University of Rochester, Rochester, NY 14627*
[3]*T.J. Watson Laboratory of Applied Physics, California Institute of Technology, Pasadena, California 91125, USA*

In this supplement detailed information is provided on the following: design of the pulley waveguide, design of the driving electrodes, discussion on the mechanism of electro-optic modulation of the soliton repetition rate, estimation of fequency modulation amplitude, and frequency modulation at frequencies beyond the photon lifetime limit of the resonator.

A. Design of the pulley coupling waveguide

To prevent the Raman lasing, we design the pulley coupling waveguide such that the resonator is close to critical coupling around the pump wavelength of 1550 nm but it is strongly over-coupled at the Raman Stokes wavelength around 1720 nm. The effective refractive index of the waveguides is modeled by the finite-element method via COMSOL, and the coupling condition of the bus waveguide can be simulated with a coupled-mode theory [1]. Our detailed modelings show that the desired coupling condition can be obtained with a bus-waveguide width of 1.765 µm, a pulley angle of 10 degrees, and a constant gap of 300 nm between the bus waveguide and the ring resonator in the pulley coupling region. Figure S1 shows the simulated ratio of the external coupling Q, Q_{ex}, to the intrinsic optical Q, Q_0. It shows that such a pulley waveguide design is able to achieve nearly critical coupling at the pump wavelength of 1550 nm but is strongly over coupled at the Raman Stokes wavelength around 1720 nm. A side effect of such a design is that a coupling resonance appears around a wavelength of 1580 nm around which the resonator is deeply under coupled. This side effect is responsible for the spectral distortion of the soliton combs shown in Fig.2 (c) and (f) of the main text. The side effect can be removed by further optimizing the pulley waveguide design. A similar approach was used in Ref. [2].

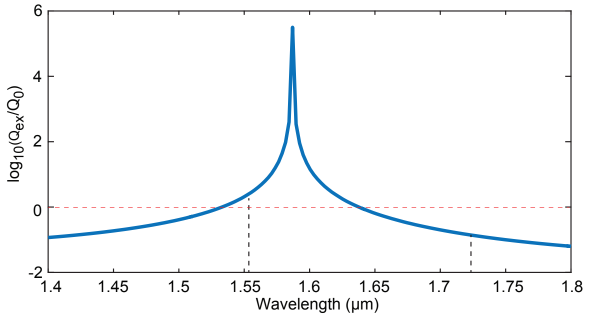

FIG. S1. Simulated ratio of external coupling quality factor Q_{ex} to intrinsic quality factor Q_0 for the designed pulley bus waveguide.

B. Design of electro-optic tuning/modulation element for the soliton comb resonator

A general theory of the electro-optic effect in an LN electro-optic resonator: Here we provide first a general theory describing the electro-optical (EO) effect in an LN electro-optic resonator, and then apply it for designing the EO tuning/modulation elements for the z-cut soliton comb resonator.

In general, a driving electric field $\boldsymbol{\mathcal{E}} = \mathcal{E}_i$ applied to the LN medium will perturb the relative permittivity tensor $\boldsymbol{\epsilon}_r = (\epsilon_r)_{ij}$ and introduce a change $\delta\boldsymbol{\epsilon}_r = (\delta\epsilon_r)_{ij}$,

$$(\delta\epsilon_r)_{ij} = -(\epsilon_r)_{ik} r_{klm} \mathcal{E}_m (\epsilon_r)_{lj}. \quad (1)$$

To obtain Eq. (1), we have used the electro-optic Pockels tensor $r_{ijk} \equiv (\partial\eta_{ij}/\partial\mathcal{E}_k)_{\mathcal{E}=0}$ where η_{ij} is the impermeability tensor which is related to the relative permittivity tensor $(\epsilon_r)_{ij}$ as $\eta_{ij} \equiv (\epsilon_r)^{-1}_{ij}$ [3]. In the crystallographic coordinate system of LN, the relative permittivity tensor is given by

$$(\epsilon_r)_{ij} = \begin{pmatrix} \epsilon_{11} & 0 & 0 \\ 0 & \epsilon_{11} & 0 \\ 0 & 0 & \epsilon_{33} \end{pmatrix}, \quad (2)$$

where ϵ_{11} and ϵ_{33} are the relative permittivity coefficients in Cartesian coordinates, and the z-axis corresponds to the optic axis of LN. Accordingly, the Pockels tensor r_{ijk} has a contracted form r_{ij} [3]:

$$r_{ij} = \begin{pmatrix} 0 & -r_{22} & r_{13} \\ 0 & r_{22} & r_{13} \\ 0 & 0 & r_{33} \\ 0 & r_{51} & 0 \\ r_{51} & 0 & 0 \\ -r_{22} & 0 & 0 \end{pmatrix}. \quad (3)$$

* These two authors contributed equally.
† Electronic mail: qiang.lin@rochester.edu

The EO induced perturbation to the dielectric tensor affects the cavity resonance ω_0 by introducing a shift $\delta\omega_0$, which can be obtained via perturbation theory [4, 5]:

$$\delta\omega_0 = -\frac{\omega_0}{2}\frac{\langle \mathbf{E}^\dagger \delta\boldsymbol{\epsilon}_r \mathbf{E}\rangle}{\langle \mathbf{E}^\dagger \boldsymbol{\epsilon}_r \mathbf{E}\rangle} = -\frac{\omega_0}{2}\frac{\langle E_i, (\delta\epsilon_r)_{ij} E_j\rangle}{\langle E_i, (\epsilon_r)_{ij} E_j\rangle}, \tag{4}$$

where $\mathbf{E} = E_i$ is the optical field vector of the cavity resonance mode. By using Eqs. (2) and (3) in Eq. (1) and then substituting it into Eq. (4), we obtain the resulting EO-induced cavity resonance shift as

$$\delta\omega_0 = -\frac{\omega_0}{2}\frac{\int_{\rm core} \mathrm{d}^3\mathbf{r} \sum_{u,v} \xi_u^{(v)}}{\int_{\rm all} \mathrm{d}^3\mathbf{r}\bar{\xi}}, \quad u \in \{x, y, z\},\ v \in \{1, 2\}, \tag{5}$$

where $\xi_u^{(v)}$ represents the fractional contribution from different polarization components given as:

$$\xi_x^{(1)} = 2\mathcal{E}_x r_{51}\epsilon_{33}\epsilon_{11}\,\mathrm{Re}\{E_x E_z^*\}, \tag{6}$$

$$\xi_x^{(2)} = -2\mathcal{E}_x r_{22}\epsilon_{11}^2\,\mathrm{Re}\{E_x E_y^*\}, \tag{7}$$

$$\xi_y^{(1)} = \mathcal{E}_y r_{22}\epsilon_{11}^2\left(|E_y|^2 - |E_x|^2\right), \tag{8}$$

$$\xi_y^{(2)} = 2\mathcal{E}_y r_{51}\epsilon_{33}\epsilon_{11}\,\mathrm{Re}\{E_y E_z^*\}, \tag{9}$$

$$\xi_z^{(1)} = \mathcal{E}_z r_{13}\epsilon_{11}^2\left(|E_x|^2 + |E_y|^2\right), \tag{10}$$

$$\xi_z^{(2)} = \mathcal{E}_z r_{33}\epsilon_{33}^2\,|E_z|^2, \tag{11}$$

where E_x ($\mathcal{E}_x$), E_y ($\mathcal{E}_y$), and E_z ($\mathcal{E}_z$) are the three polarization components of the optical field vector $\mathbf{E}$ ($\boldsymbol{\mathcal{E}}$). $\bar{\xi}$ is the normalization factor for the optical field given as

$$\bar{\xi} = \epsilon_{11}\left(|E_x|^2 + |E_y|^2\right) + \epsilon_{33}\,|E_z|^2, \tag{12}$$

that is related to the energy density of the optical mode. In Eq. (5), $\int_{\rm core}$ stands for the spatial integration over the LN waveguide layer only, and $\int_{\rm all}$ stands for integration over the whole space. In $\xi_u^{(v)}$ (Eqs. (6)-(11)), the subscript u ($u = x, y, z$) denotes the contribution from the u polarization component of the driving electric field and the superscript index v ($v = 1, 2$) denotes the contribution from different polarization components of the optical cavity mode.

Equations (5)-(12) can be used to describe the electro-optic effect in any arbitrary LN electro-optic micro/nanoresonator, including microring, racetrack, microdisk, and photonic crystals.

Design of EO tuning/modulation elements for the z-cut soliton comb resonator: We now use the theory developed above to describe the electro-optic effect in a z-cut microring resonator. Specifically, we are interested in the EO effect on the fundamental quasi-TE modes of a circularly shaped z-cut microring resonator that we employ for producing soliton microcomb.

Figure S2 shows the optical mode field profile of a fundamental quasi-TE mode in such a resonator. It shows clearly that the cavity mode has its polarization dominantly lying in the device plane (the x-y plane) along the radial direction, with E_z component negligible. The optical field $\mathbf{E}$ can thus be approximated as:

$$\mathbf{E} \approx E_0(r, z)\left(\cos\theta\hat{x} + \sin\theta\hat{y}\right)e^{im\theta}, \tag{13}$$

where $E_0(r, z)$ is the optical field amplitude, m is the mode number of the cavity mode, and $\hat{x}$ ($\hat{y}$) is the unit vector in the x (y) axis. Due to the rotational symmetry of device, we have adopted a cylindrical coordinate system (r, θ, z) in Eq. (13).

For the EO tuning and modulation, we focus on the scenario that the electrodes are placed on the opposite sides of the ring waveguide (Fig. S2), which is easy to implement in practice. For this case, as shown in Fig. S2, the driving electric field inside the LN waveguide core also dominantly lies in the x-y plane and along the radial direction. As such, the driving electric field vector $\boldsymbol{\mathcal{E}}$ can be approximated as

$$\boldsymbol{\mathcal{E}} \approx \mathcal{E}_0(r, z)\left(\cos\theta\hat{x} + \sin\theta\hat{y}\right), \tag{14}$$

where $\mathcal{E}_0(r, z)$ is the driving field amplitude.

With the optical field and driving electric field given in Eqs. (13) and (14), Eqs. (6)-(11) show that, among all the terms, only $\xi_x^{(2)}$ and $\xi_y^{(1)}$ are non-negligible, and these are given by

$$\xi_x^{(2)} = -r_{22}\epsilon_{11}^2\mathcal{E}_0(r, z)|E_0(r, z)|^2\sin(2\theta)\cos\theta, \tag{15}$$

$$\xi_y^{(1)} = -r_{22}\epsilon_{11}^2\mathcal{E}_0(r, z)|E_0(r, z)|^2\sin\theta\cos(2\theta). \tag{16}$$

As a result, the EO-induced cavity resonance shift (Eq. (5)) becomes

$$\begin{aligned}\delta\omega_0 &= \frac{\omega_0}{4\pi}\frac{\int_{\rm core} r_{22}\epsilon_{11}^2\mathcal{E}_0|E_0|^2 r\,\mathrm{d}r\,\mathrm{d}z \int_{\theta_1}^{\theta_2}\sin(3\theta)\,\mathrm{d}\theta}{\int_{\rm all}\epsilon_{11}|E_0|^2 r\,\mathrm{d}r\,\mathrm{d}z} \\ &= \frac{\omega_0}{4\pi}\frac{\int_{\rm core} r_{22}\epsilon_{11}^2\mathcal{E}_0|E_0|^2 r\,\mathrm{d}r\,\mathrm{d}z}{\int_{\rm all}\epsilon_{11}|E_0|^2 r\,\mathrm{d}r\,\mathrm{d}z}\frac{1}{3}\left[\cos(3\theta_1) - \cos(3\theta_2)\right],\end{aligned} \tag{17}$$

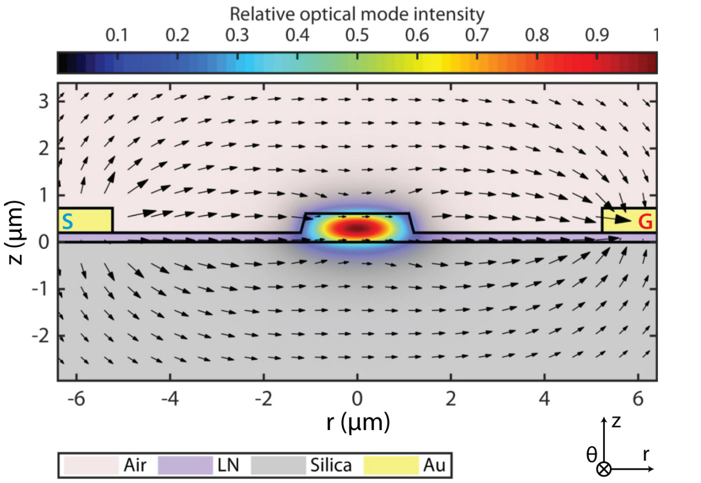

FIG. S2. Optical mode field profile (color map) of the fundamental quasi-TE mode and vector map of applied electric field (arrow map) of the LN microring comb resonator, simulated by the finite-element method.

where we have assumed that the driving electrodes span over an azimuthal angle range between θ_1 and θ_2. Apparently, a driving electrode spanning over the entire circular ring would result in a zero net frequency shift. However, Eq. (17) shows that $\delta\omega_0$ exhibits a period of 120° and it reaches a peak value by choosing $(\theta_1, \theta_2) = (0, 60^o)$. Moreover, $\delta\omega_0$ remains at the same peak value for $(\theta_1, \theta_2) = (0, 60^o)$, $(120^o, 180^o)$, or $(240^o, 300^o)$ but flips its sign for $(\theta_1, \theta_2) = (60, 120^o)$, $(180^o, 240^o)$, or $(300, 360^o)$. Therefore, we can maximize the magnitude of the EO-induced resonance frequency shift by alternating the sign or polarity of the driving field across six identical electrode pairs placed consecutively along the microring circumference. Figure S3 shows the arrangement of the driving electrodes. With this approach, the induced frequency shifts by the six electrode sections all add up constructively, resulting in a maximal EO frequency shift of

$$\delta\omega_0 = \frac{\omega_0}{\pi} \frac{\int_{\text{core}} r_{22}\epsilon_{11}^2 \mathcal{E}_0 |E_0|^2 r \, \text{d}r \, \text{d}z}{\int_{\text{all}} \epsilon_{11} |E_0|^2 r \, \text{d}r \, \text{d}z}. \tag{18}$$

To verify the function of the proposed EO modulation structure shown in Fig. S3 and to quantify the magnitude of resulting EO tuning, we used the full vectorial form of the optical field and that of the driving electric field simulated by the finite-element method, in Eqs. (5)-(12) to find the induced resonance frequency shift. The results are presented in Fig. S4, which show the dependence of $\Delta\nu = \delta\omega_0/(2\pi)$ on both the electrode coverage angle θ and the waveguide-electrode spacing $d_{\text{w-e}}$, with an applied voltage of 1 V. Figure S4(b) shows that, for a waveguide-electrode spacing of 4 µm, each pair of signal-ground driving electrodes offers a frequency tuning efficiency of ∼12 MHz/V. As a result, a group of two-pairs of driving electrodes, as built into our device, will produce a frequency tuning efficiency of ∼24MHz/V, corresponding to a wavelength tuning efficiency of ∼0.19 pm/V which is close to what we measured on the fabricated device. In particular, the driving electrode structure proposed in Fig. S3 does offer constructive EO tuning effect from the six pairs of the electrodes, with $\Delta\nu$ increasing with the azimuthal angle up to a value of ∼72 MHz/V. As shown in Fig. S4(a) and (c), the EO tuning efficiency increases with decreased waveguide-electrode spacing, reaching a value of ∼130 MHz/V for $d_{\text{w-e}} \sim 2$ µm. However, our experimental testing shows that such a small $d_{\text{w-e}}$ would degrade the intrinsic optical Q of the resonator due to the perturbation of electrodes to the optical mode. To prevent such impact, we use a $d_{\text{w-e}}$ value of 4 µm in the fabricated devices, which will leave the optical Q intact.

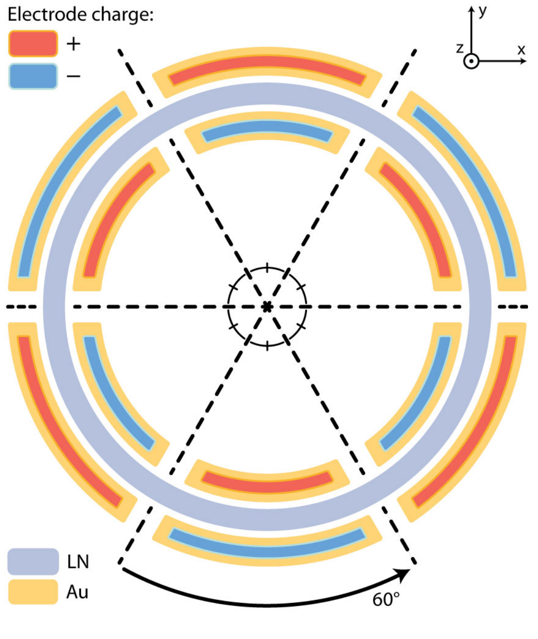

FIG. S3. Layout of the driving electrodes for a z-cut microring resonator. There are six pairs of electrodes with alternating polarities all arranged symmetrically and spaced azimuthally by 60° along the ring circumference. Colors indicate Lithium niobate (LN), gold (Au), or electrode charge (+ or −).

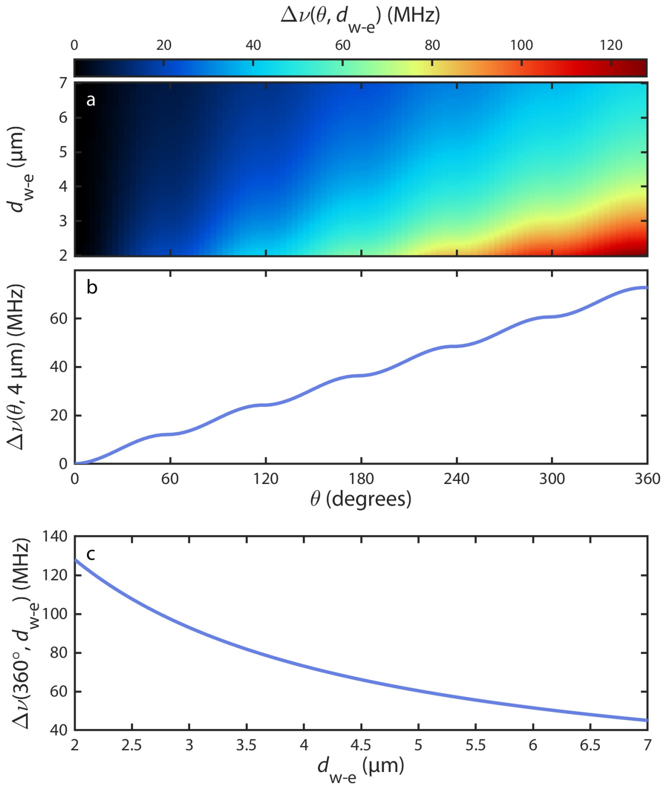

FIG. S4. Simulated frequency shift $\Delta\nu$ vs. azimuthal angle θ and waveguide-electrode spacing $d_{\text{w-e}}$ with an applied voltage of 1 V, for a resonance frequency of ∼ 193.4 THz (1.55 µm). Top: surface plot vs. θ and $d_{\text{w-e}}$ where the colormap represents the frequency shift $\Delta\nu$. Middle: $\Delta\nu$ vs. electrode coverage angle θ for $d_{\text{w-e}} \sim 4$ µm. Bottom: $\Delta\nu$ vs. waveguide-electrode spacing $d_{\text{w-e}}$ with a full 2π microring electrode coverage.

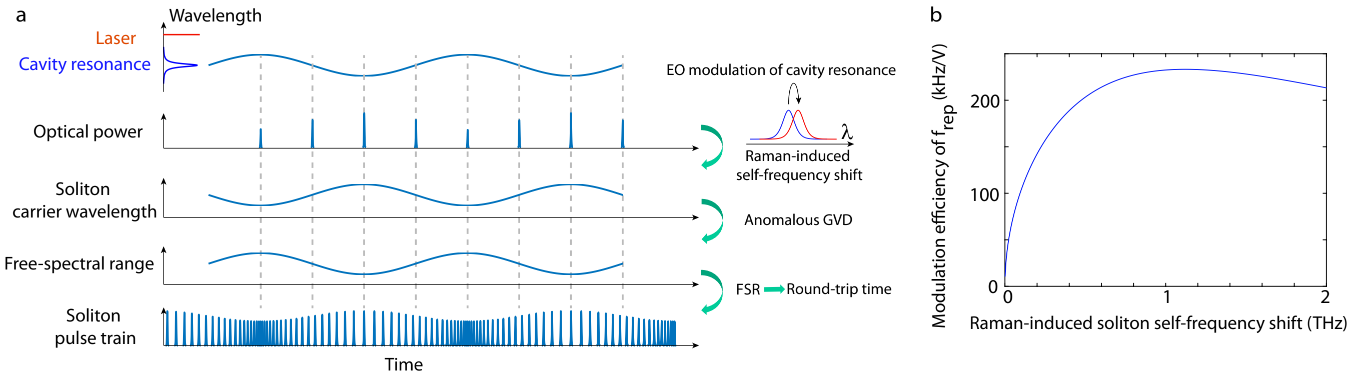

FIG. S5. (a) Schematic showing the underlying mechanism of EO tuning soliton repetition rate. (b) Theoretical FM efficiency as a function of Raman-induced SFS of soliton. The device parameters same as Fig. 3 of the main text are used in the theoretical calculation, with the loaded optical Q of 3.2 million, group index $n_g = 2.357$, FSR = 19.81 GHz. $\tau_R = 6.3$ fs is adopted from Ref. [6].

C. On the mechanism of EO modulation of the soliton repetition rate

As discussed in the main text, one potential mechanism responsible for the observed FM of the microwave signal is the Raman-induced SFS of the solitons whose magnitude depends on the laser-cavity detuning [7, 8]. EO modulation of the comb resonator modulates the laser-cavity detuning of the pump wave which in turn changes the magnitude of SFS and thus shifts the carrier frequency of the Kerr solitons. Due to the group-velocity dispersion of the resonator, such a shift of soliton carrier frequency translates into a change of the repetition rate. Figure S5(a) illustrates the overall mechanism.

The repetition rate of the Kerr solitons is determined by the free-spectral range (FSR) of the resonator at the carrier frequency of the solitons. Due to the group-velocity dispersion of the resonator, FSR is generally frequency dependent. As a result, a shift of the soliton carrier frequency, $\delta\Omega$, would translate into a change of FSR, δ(FSR) given by the following expression

$$\frac{\delta(\mathrm{FSR})}{\mathrm{FSR}} = -v_g \beta_2 \delta\Omega, \tag{19}$$

where v_g and β_2 are the group velocity and group-velocity dispersion, respectively. The carrier frequency of soliton is impacted by the Raman-induced self-frequency shift, Ω_R, which is related to the laser-cavity detuning of the pump wave, Δ, as [7]

$$\Delta = A\sqrt{\Omega_R} - B\Omega_R^2, \tag{20}$$

where $\Delta = \omega_0 - \omega_p$ is the frequency detuning between the cavity resonance ω_0 and the pump laser frequency ω_p. $A \equiv \sqrt{\frac{15c|\beta_2|\omega_0}{32 n_0 Q \tau_R}}$ and $B \equiv \frac{c\beta_2}{2n_0}$ where c is the velocity of light in vacuum. n_0 and τ_R are the refractive index and the Raman time constant of LN, respectively. Q is the loaded optical Q of the device. A small change of the laser-cavity detuning, $\delta\Delta$, would lead to a small change of SFS of the soliton, $\delta\Omega_R$, given by

$$\delta\Omega_R = \frac{\delta\Delta}{\frac{A}{2\sqrt{\Omega_R}} - 2B\Omega_R}. \tag{21}$$

EO modulation of the comb resonator would result in time-dependent modulation of the cavity resonance $\delta\omega_0(t) = \eta V_p \cos(\Omega_m t) = \delta\Delta(t)$, where η is the EO tuning efficiency, V_p is the peak value of the driving voltage, and Ω_m is the modulation frequency. Using this expression in Eq. (21) and then applying it in Eq. (19), we thus obtain the time-dependent modulation on the FSR given by

$$\frac{\delta(\mathrm{FSR})}{\mathrm{FSR}} = \frac{-v_g \beta_2 \eta V_p \cos(\Omega_m t)}{\frac{A}{2\sqrt{\Omega_R}} - 2B\Omega_R}. \tag{22}$$

As a result, the FM efficiency of the microwave, defined as the FSR change per unit driving voltage, is given by the following expression

$$\rho_{\mathrm{FM}} = \frac{-v_g \beta_2 \eta \mathrm{FSR}}{\frac{A}{2\sqrt{\Omega_R}} - 2B\Omega_R}. \tag{23}$$

Figure S5 (b) shows the calculated theoretical FM efficiency of our device. Due to the spectral distortion induced by the coupling waveguide (Fig. 2 in the main text), it is difficult to quantify the amount of SFS of the soliton combs. As an estimate, it is generally in the range of (1-2) THz [6]. As shown in Fig. S5 (b), the induced FM efficiency is roughly in the range of (100–200) kHz/V. This level of FM efficiency explains well the observed phenomena at a low modulation frequency. However, it is significantly lower than we observed at a high modulation frequency of 50 and 75 MHz. The underlying mechanism is not clear at this moment and will require further exploration.

D. Estimation of FM amplitude at a high modulation frequency

The electric spectrum analyzer we employed (Tektronix, RSA5126B) has a limited bandwidth of 160 MHz for time-dependent frequency characterization (although it does not have such bandwidth limitation when it is used for spectrum analysis). So for a high modulation frequency such as 75 MHz, it can only capture the first-order modulation sidebands. However, the spectrum of the microwave (Fig. 3(e) of the main text) shows multiple orders of modulation sidebands, implying that the spectrogram analysis likely underestimates the amplitude of frequency modulation (FM). Here we use the spectrum of the microwave for a rough estimate of the FM amplitude.

The theory in the previous section shows that the modulation of soliton repetition rate results from the Raman-induced SFS via the modulation of soliton energy [7]. As a result, it is expected that the frequency modulation (FM) of the detected microwave will be accompanied with a certain extent of amplitude modulation (AM). In the time domain, an electric field E of a microwave that is undergoing time-varying phase modulation as well as amplitude modulation can be expressed as follows:

$$E(t) = \frac{E_0}{\sqrt{1+a^2}}[1 + a\sin(\Omega_m t)]e^{i(\Omega_0 t + \sigma\cos\Omega_m t)}, \quad (24)$$

where E_0 is the field amplitude, Ω_0 is the carrier frequency of the microwave, and Ω_m is the modulation frequency. σ and a are the modulation depths of phase modulation and amplitude modulation, respectively. The microwave field shown in Eq. (24) exhibits an instantaneous frequency as

$$\Omega(t) = \Omega_0 - \sigma\Omega_m \sin\Omega_m t, \quad (25)$$

which varies with time in a sinusoidal fashion with an FM amplitude of $\sigma\Omega_m$. In Eqs. (24) and (25), we have assumed that the FM and AM are out of phase, which is expected from the Raman-induced SFS effect of the soliton: The higher the soliton energy (and thus the larger the amplitude of the detected microwave), the larger the induced SFS and thus the longer the carrier wavelength of the soliton. The longer the carrier wavelength, the lower the repetition rate (and thus the lower the microwave frequency) due to anomalous GVD of the device.

Equation (24) can be expanded in terms of the Bessel functions as

$$\begin{aligned} E(t) &= \frac{E_0 e^{i\Omega_0 t}}{\sqrt{1+a^2}}[1 + a\sin(\Omega_m t)] \sum_{n=-\infty}^{\infty} i^n J_n(\sigma) e^{in\Omega_m t} \\ &= \frac{E_0 e^{i\Omega_0 t}}{\sqrt{1+a^2}} \sum_n i^n e^{n\Omega_m t}\left[J_n - \frac{a}{2}J_{n-1} - \frac{a}{2}J_{n+1}\right], \end{aligned} \quad (26)$$

where $J_n(\sigma)$ is the nth order Bessel function of the first kind. High-speed phase modulation (frequency modulation) produces a series of modulation sidebands whose

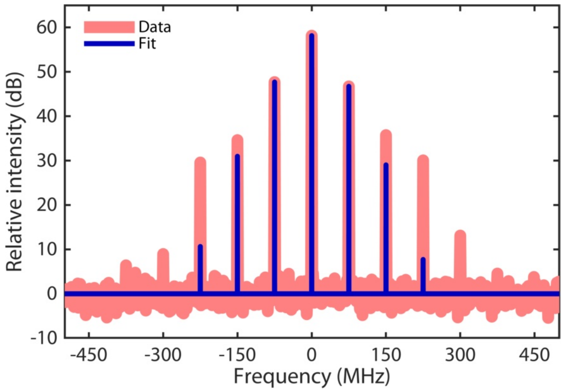

FIG. S6. Comparison between the theoretical and experimentally observed spectra of the 19.81 GHz microwave with a modulation frequency of 75 MHz. Red line: experimentally recorded RF spectrum from Fig.3 (e) of the main text. Blue line: theoretically calculated spectrum, with $\sigma = 0.55$ and $a = 0.03$.

frequencies are separated from the carrier frequency by an integer number of the modulation frequency with amplitudes scaled with the Bessel function coefficients $J_n(\sigma)$ whose magnitude depends on the modulation depth σ. We can thus use this approach to provide a rough estimate on the modulation depth, by comparing the theoretical spectrum with the experimentally recorded one. Figure S6 shows the results for the modulation frequency of 75 MHz. A phase-modulation depth of $\sigma = 0.55$ and an amplitude modulation depth of $a = 0.03$ provide a reasonably good description of the modulation sidebands up to the first two orders whose relative amplitudes agree closely the experimental ones. Accordingly, the FM amplitude, $\sigma\Omega_m/(2\pi)$, is estimated to be ~ 41 MHz. Figure S6 shows certain discrepancies on the higher-order sidebands between the theoretical estimation and experiment data, which likely implies a certain distortion associated with the modulation.

It is important to note that such a brief spectrum analysis can only provide a rough estimate of the level of frequency modulation, since the relative phases of the modulation sidebands play crucial roles on the real extent of FM, which cannot be obtained from the spectrum. The exact extent of FM/AM will require larger characterization bandwidth of the spectrum analyzer, which cannot be perform at this moment and will be left for future exploration.

E. Modulation of soliton repetition rate at a speed beyond the photon lifetime limit of the comb resonator

The driving electrodes of the device support modulation at a speed beyond the photon lifetime limit of the resonator. At such a high speed, however, the employed spectrum analyzer cannot perform the time-dependent

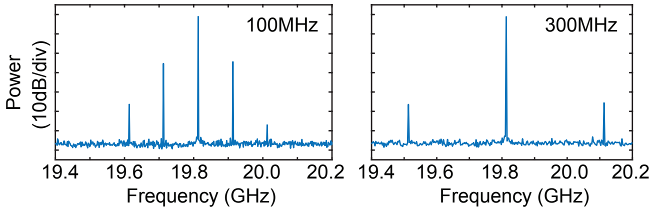

FIG. S7. Microwave spectra for the modulation frequency at 100 MHz (left) and 300 MHz (right), with a peak driving voltage similar to the case of 75 MHz shown in the main text.

frequency analysis due to its bandwidth limit (160 MHz). However, it is still able to perform spectrum characterization of the microwave signal. Figure S7 shows two examples with a modulation frequency of 100 and 300 MHz, respectively. Clearly, since the modulation frequency is considerably beyond the photon lifetime limit of the comb resonator, the modulation efficiency drops considerably, as evident by the smaller amplitudes of the modulation sidebands, compared to the case of 75 MHz shown in the main text.

[1] G. Moille, Q. Li, T. C. Briles, S.-P. Yu, T. Drake, X. Lu, A. Rao, D. Westly, S. B. Papp, and K. Srinivasan, Optics letters **44**, 4737 (2019).

[2] Z. Gong, M. Li, X. Liu, Y. Xu, J. Lu, A. Bruch, J. B. Surya, C. Zou, and H. X. Tang, Physical Review Letters **125**, 183901 (2020).

[3] A. Yariv and P. Yeh, *Optical waves in crystals*, Vol. 5 (Wiley New York, 1984).

[4] J. D. Joannopoulos, S. G. Johnson, J. N. Winn, and R. D. Meade, in *Photonic Crystals* (Princeton university press, 2011).

[5] S. G. Johnson, M. Ibanescu, M. Skorobogatiy, O. Weisberg, J. Joannopoulos, and Y. Fink, Physical review E **65**, 066611 (2002).

[6] Y. He, Q.-F. Yang, J. Ling, R. Luo, H. Liang, M. Li, B. Shen, H. Wang, K. Vahala, and Q. Lin, Optica **6**, 1138 (2019).

[7] X. Yi, Q.-F. Yang, K. Y. Yang, and K. Vahala, Optics letters **41**, 3419 (2016).

[8] C. Bao, Y. Xuan, C. Wang, J. A. Jaramillo-Villegas, D. E. Leaird, M. Qi, and A. M. Weiner, Opt. Lett. **42**, 759 (2017).
